# 针吸活检对肺癌伴肺门纵隔淋巴结转移的诊断价值

**DOI:** 10.3779/j.issn.1009-3419.2010.05.13

**Published:** 2010-05-20

**Authors:** 嵩 张, 洪莉 伊, 淑娟 姜, 元堂 李, 永康 王

**Affiliations:** 1 250021 济南，山东大学附属省立医院呼吸内科 Department of Respiratory Medicine, Provincial Hospital Affiliated to Shandong University, Jinan 250021, China; 2 250021 济南，山东大学附属省立医院细胞室; 3 250021 济南，山东大学附属省立医院病理科

**Keywords:** 经支气管针吸活检, 经支气管超声引导针吸活检, 淋巴结, Transbronchial needle aspiration, Endobronchial ultrasound-guided transbronchial needle aspiration, Lymph nodes

## Abstract

**背景与目的:**

经支气管针吸活检（transbronchial needle aspiration, TBNA）和经支气管超声引导针吸活检（endobronchial ultrasound-guided TBNA, EBUS-TBNA）是用于诊断纵隔淋巴结病变的最新检查方法。本研究旨在评价TBNA和EBUS-TBNA对肺癌伴肺门纵隔淋巴结转移的诊断价值及安全性。

**方法:**

对CT检查疑似肺癌但管腔内无明显新生物且肺门纵隔淋巴结肿大的250例患者行TBNA后行活检或刷检，15例疑似肺癌患者行EBUS-TBNA。

**结果:**

180例患者TBNA、刷检和活检确诊为肺癌，阳性率分别为82.86%、51.24%和45.45%。15例EBUS-TBNA的阳性率为91.67%。

**结论:**

TBNA和EBUS-TBNA检查安全性好，准确率高，对肺癌伴肺门纵隔淋巴结转移的患者有较高的诊断价值。

## Abstract (en)

**Background and objective:**

Transbronchial needle aspiration (TBNA) and endobronchial ultrasoundguided TBNA (EBUS-TBNA) have been applied to the diagnosis for mediastinal lymph nodes. The aim of this study is to evaluate the clinical value and safety of TBNA and EBUS-TBNA on hilar and mediastinal lymph nodes of lung cancer patients.

**Methods:**

Two hundred fifty patients with suspected lung cancer were enrolled. All petients with hilar and/or mediastinal lymphoadenopathy found by CT scan received TBNA, biopsy and brushing. EBUS-TBNA was performed in 15 patients among them.

**Results:**

Lung cancer were confirmed in 180 patients by TBNA, biopsy and brushing. The positive rates were 82.86%, 51.24% and 45.45%. Fifteen patients after EBUS-TBNA had a positive rate of 91.67%.

**Conclusion:**

TBNA and EBUS-TBNA were proved to be safe procedure with a high yield for the diagnosis of hilar and mediastinal lymph nodes in lung cancer patients.

支气管镜检查是诊断肺癌的重要手段之一，大多数肺内占位性病变可通过常规气管镜下活检或刷检获得标本进行病理学、细胞学检查而明确诊断。但对于官腔内无明显新生物的肺内占位性病变以及仅表现肺门纵隔淋巴结肿大的患者，常规活检和刷检难以确诊。经支气管针吸活检（transbronchial needle aspiration, TBNA）和经支气管超声引导针吸活检（endobronchial ultrasound-guided TBNA, EBUS-TBNA）是支气管镜的辅助检查方法。2005年1月-2010年3月，我科对250例CT检查疑似肺癌且肺门纵隔淋巴结肿大患者行TBNA检查，并根据实际情况行活检或刷检。另对15例患者行EBUS-TBNA检查，现总结报道如下。

## 1 材料与方法

### 1.1 一般资料

2005年1月-2010年3月于山东省立医院就诊的疑似肺癌患者250例，其中纵隔淋巴结肿大225例，纵隔淋巴结肿大伴单侧或双侧肺门淋巴结肿大25例，男176例，女74例；年龄15岁-84岁，中位年龄47岁。另有15例患者行EBUS-TBNA，男11例，女4例；年龄38岁-64岁，中位年龄42岁。所有患者术前心电图、血常规、凝血常规、乙肝五项均未发现异常。均签署知情同意书。

### 1.2 方法

#### 1.2.1 TBNA

250例经痰细胞学检查、常规支气管镜检查未发现明显病变或经皮肺活检等检查未获得明确诊断，胸部CT检查发现肺门或纵隔有肿大淋巴结患者行TBNA检查。根据胸部薄层强化CT扫描结果，按照WANG氏定位法^[1]^确定穿刺部位、角度和深度。纤支镜经鼻孔进入气管隆突，观察腔内有无可视性病变。穿刺针将沿气管镜的活检通道进入气道，调整气管镜，将穿刺针固定于穿刺部位，使其与气管壁接近90°角，用力将穿刺针刺入预定位置软骨环间的气管粘膜，采用推进法结合猛刺法、咳嗽法穿透气管壁刺入纵隔淋巴结，镜下看到穿刺针完全刺入气管壁内（刺入深度1.0 cm -1.5 cm）时拔出针芯将60 mL注射器连接在穿刺针尾端，抽吸至40 mL容积并持续30 s-40 s维持负压，同时，操作者在穿刺针不脱出气道粘膜的情况下，从不同方向来回抽动穿刺针以增加获取标本的机率。解除负压，拔除穿刺针，将穿刺物直接涂片，迅速送细胞学检查。所有患者于TBNA后常规刷检或活检。

#### 1.2.2 EBUS-TBNA

15例患者经口插入内镜，将内镜探头固定于穿刺部位，开启超声检查，确定穿刺病灶及穿刺距离。开启多普勒血流检查，再次确定穿刺部位。开启超声内镜，充盈水囊，以水囊紧贴穿刺部位，进行穿刺。余同TBNA。

#### 1.2.3 TBNA和EBUS-TBNA结果判断

细胞学涂片中如可见多个淋巴细胞团，认为穿刺成功；涂片中如淋巴细胞较少且见大量纤毛柱状细胞则认为穿刺失败。细胞学涂片中见到明确的恶性肿瘤细胞，即使不能区别类型或分化程度，均认为TBNA或EBUS-TBNA结果阳性；细胞学涂片中如仅见淋巴细胞，认为TBNA或EBUS-TBNA结果阴性。

### 1.3 统计学处理

应用SPSS 13.0行t或χ^2^检验。

## 2 结果

### 2.1 气管镜检查结果

263例患者（2例患者先后行TBNA和EBUS-TBNA）纤维支气管镜下均未见明显新生物，122例常规气管镜检查正常，98例局部支气管粘膜充血、增厚、粗糙、糜烂或有小结节样改变，43例管腔呈外压型改变。

### 2.2 临床诊断结果

250例患者共有210例最终确诊为肺癌。各种取材方法的阳性率情况见表 1。共有180例患者经气管镜检查获得明确诊断，其余30例确诊患者2例由EBUS-TBNA确诊，2例由纵隔镜确诊，1例患者有浅表淋巴结肿大，穿刺活检证实为肺癌，25例由外科手术明确。

**1:** 210例肺癌患者经气管镜不同取材方法诊断情况 The diagnosis results of different methods in 210 lung cancer patients

Methods	Positive patients	Total patients	Positive rate
Brushing	62	121	51.24%
Biopsy	45	99	45.45%
Brushing+Biopsy	85	133	63.91%
TBNA	174	210	82.86%
Brushing+Biopsy+TBNA	180	210	85.71%
TBNA: transbronchial needle aspiration.			

### 2.3 TBNA并活检、刷检结果

174例患者TBNA检查为阳性，其中男132例，女42例。非小细胞肺癌68例，小细胞肺癌46例，另有60例查到癌细胞但未明确分型。TBNA的阳性率为82.86%（174/210）。TBNA细胞学诊断见图 1。有62例患者刷检阳性，其中非小细胞肺癌36例，小细胞肺癌26例，有6例患者刷检阳性而TBNA检查阴性。6例患者均为男性，非小细胞肺癌和小细胞肺癌各3例。常规刷检的阳性率为51.24%（62/121）。由于250例患者均无明显腔内占位性病变，仅45例（45.45%）获得组织病理学诊断，其中小细胞肺癌16例，鳞癌12例，腺癌7例，上皮内肿瘤1例（TBNA检查阳性），涎腺型肿瘤（TBNA检查示非小细胞肺癌）1例，病理类型不明确者8例。TBNA对肺癌的诊断率明显高于刷检和活检。

**1:**
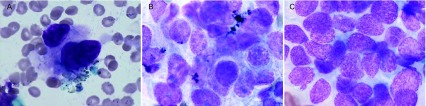
肺癌细胞学诊断。A：鳞癌；B：腺癌；C：小细胞癌。 Cytopathology diagnosis of lung cancer. A: squamous cell carcinoma; B: adenocarcinoma; C: small cell lung carcinoma.

### 2.4 不同部位淋巴结TBNA检查结果

由表 2可知，前隆突淋巴结、右气管旁淋巴结、隆突下淋巴结和隆突远端淋巴结穿刺获得阳性诊断较多。TBNA对外压性病变有较高的诊断率，达74.42%（32/43），如合并淋巴结肿大可进一步提高诊断率。

**2:** 不同的穿刺部位淋巴结TBNA检查的情况 The diagnosis results of different puncture site by TBNA

Puncture site	Positive patients
Anterior carina lymph node	37
Posterior carina lymph node	5
Right paratrachea lymph node	33
Left paratrachea lymph node	5
Right mainstem bronchus lymph node	7
Left mainstem bronchus lymph node	4
Right upper hilar lymph node	6
Subcarina lymph node	27
Right lower hilar lymph node	3
Sub-subcarina lymph node	17
Left hilar lymph node	9
Extrinsic compressing lesions	32

### 2.5 EBUS-TBNA

15例患者有12例最终确诊为肺癌。其中11例EBUS-TBNA阳性，阳性率为91.67%，另1例EBUSTBNA细胞学检查见较多坏死组织，未查到癌细胞，另行TBNA检查证实为小细胞肺癌。

### 2.6 并发症

部分TBNA和EBUS-TBNA穿刺部位少量出血，给予生理盐水冲洗，无需应用止血药或其他特别处理，所有患者均未出现大出血、纵隔气肿、纵隔感染等不良反应。患者均能很好耐受操作。

## 3 讨论

肺癌是最常见的恶性肿瘤，位居癌症死亡的首位^[2]^。支气管镜检查是早期肺癌特别是中心型肺癌确诊的最主要手段，提高支气管镜对肺癌的病理确诊率是非常重要的。经支气管镜活检可明确组织病理学诊断，但对于外压性病变或无气管内占位的患者诊断阳性率较低。刷检由于接触病灶面积大，易接触新鲜创面，受病变表面出血影响较小，且可进入细小支气管，较易找到癌细胞，故本组细胞病理学诊断阳性率略高于活检。支气管镜下主要表现为外压性改变的肺癌患者通过常规的活检、刷检难以确诊，而单纯纵隔、肺门淋巴结肿大以及胸膜小结节病变更是常规支气管镜检查的盲区。对于肺门、纵隔淋巴结肿大患者的诊断和肺癌分期，纵隔镜是“金标准”^[3, 4]^。但纵隔镜因为创伤大、费用高、检查范围相对较小且需要全身麻醉或局麻下进行检查，不易被患者及家属所接受，难以作为常规的方法和手段在临床开展。

与纵隔镜相比，TBNA能突破气道限制，可通过穿刺针通过支气管镜进入气道内穿透气管壁对腔外病变（肿块、淋巴结等）进行穿刺抽吸获取细胞学或组织学标本，减少了患者的痛苦和手术风险，能大大提高诊断率，同时对表现为粘膜下病变、肺周围结节和肿块的支气管内病变也可较方便取材^[5]^。本组210例肺癌患者中有174例由TBNA确诊，阳性率为82.86%。TBNA和活检、刷检结合可进一步提高诊断率（85.71%）。TBNA纵隔检查不仅丰富和延伸了纤支镜的应用范围，而且可以明确诊断，使肺癌得分期更准确，对进一步的治疗具有指导作用。我们的结果显示前隆突淋巴结、右气管旁淋巴结、隆突下淋巴结和隆突远端淋巴结穿刺获得阳性结果较多。上述部位穿刺较容易定位，穿刺较容易且可反复操作，也是肿瘤较易转移部位。该部位出血少，对穿刺后的活检刷检影响也较少。

TBNA具有操作简单、创伤小、并发症少等特点，临床应用较多，但因其属于“盲穿”，阳性率取决于操作者的熟练程度、淋巴结的大小和位置、细胞学诊断技术等因素，波动较大（20%-89%）^[6, 7]^。EBUS-TBNA是近年来应用于临床的新技术之一，由于具有实时超声图像显示的功能，与传统的TBNA相比，穿刺定位更加精确，显著提高了穿刺的准确性及安全性。EBUS-TBNA可显示淋巴结及病变的血供情况，也可显示周围血管结构，使操作更加安全。我科开展EBUS-TBNA时间较短，样本例数较少，其中2例TBNA阴性患者经EBUS-TBNA确诊，但也有1例EBUS-TBNA阴性患者经TBNA检查确诊，TBNA操作灵活，EBUS-TBNA定位准确，二者联合应用可进一步增加诊断的阳性率。EBUS-TBNA可获得组织病理学诊断，避免了采用纵隔镜等外科手段来明确术前分期^[8]^。Yasufuku等^[9]^对102例拟手术的肺癌患者分别行CT、PET-CT和EBUS-TBNA以评估分期。结果CT、PET-CT和EBUSTBNA在诊断纵隔和肺门淋巴结的敏感性分别为76.9%、80.0%和92.3%，特异性分别为55.3%、70.1%和100%，而诊断准确率分别为60.8%、72.5%和98.0%。另外，纵隔镜检查仅能获得右气管旁和隆突前肿大淋巴结标本，对隆突下、后和左气管旁肿大淋巴结无法进行检测。TBNA和EBUS-TBNA可减少甚至代替纵隔镜、开胸等方法在肺部、纵隔疾病的应用，并且可对纵隔内直径＜1 cm的淋巴结进行穿刺检查，且无明显并发症。

TBNA和EBUS-TBNA是一种安全有效的方法，在纵隔及肺部占位性病变的诊断和治疗中具有重要的价值，值得临床推广应用。
